# Epidemic of hypertension in Ghana: a systematic review

**DOI:** 10.1186/1471-2458-10-418

**Published:** 2010-07-14

**Authors:** William K Bosu

**Affiliations:** 1 Disease Control and Prevention Department, Ghana Health Service, P O Box KB493, Accra, Ghana; 2 Non-Communicable Disease Epidemiology Unit, Department of Epidemiology & Disease Control, School of Public Health, University of Ghana, Legon, Ghana

## Abstract

**Background:**

Hypertension is a major risk factor for many cardiovascular diseases in developing countries. A comprehensive review of the prevalence of hypertension provides crucial information for the evaluation and implementation of appropriate programmes.

**Methods:**

The PubMed and Google Scholar databases were searched for published articles on the population-based prevalence of adult hypertension in Ghana between 1970 and August 2009, supplemented by a manual search of retrieved references. Fifteen unique population-based articles in non-pregnant humans were obtained. In addition, two relevant unpublished graduate student theses from one university department were identified after a search of its 1996-2008 theses.

**Results:**

The age and sex composition of study populations, sampling strategy, measurement of blood pressure, definition of hypertension varied between studies. The prevalence of hypertension (BP ≥ 140/90 mmHg ± antihypertensive treatment) ranged from 19% to 48% between studies. Sex differences were generally minimal whereas urban populations tended to have higher prevalence than rural population in studies with mixed population types. Factors independently associated with hypertension included older age group, over-nutrition and alcohol consumption. Whereas there was a trend towards improved awareness, treatment and control between 1972 and 2005, less than one-third of hypertensive subjects were aware they had hypertension and less than one-tenth had their blood pressures controlled in most studies.

**Conclusion:**

Hypertension is clearly an important public health problem in Ghana, even in the poorest rural communities. Emerging opportunities such as the national health insurance scheme, a new health policy emphasising health promotion and healthier lifestyles and effective treatment should help prevent and control hypertension.

## Background

In spite of their high medical and economic burden, cardiovascular diseases have not been accorded the needed priority globally [1]. In sub-Saharan Africa, morbidity and mortality from cardiovascular diseases are projected to increase over the coming decades [2]. The direct healthcare costs attributable to non-optimal blood pressure in sub-Saharan Africa in 2001 was estimated at two billion US dollars [3]. In the absence of adequate control measures, the prevalence of hypertension in some African countries has increased significantly to more than 30% [4,5].

Several epidemiological studies have been conducted in Ghana over the past 60 years. A survey conducted in a village about 60 miles from Accra in 1950 found that 5.5% of the 255 village inhabitants had cardiovascular diseases [6]. Nearly one quarter of the deaths in Mamprobi, Accra over the 1975-1980 period was due to cardiovascular diseases [7]. In 1981, the Ghana Health Assessment Team estimated that cerebrovascular disease and hypertensive heart disease accounted for 7% of the total healthy years of life lost [8].

The number of reported new cases of hypertension in outpatient public health facilities in Ghana increased more than ten-fold from 49,087 in 1988 to 505,180 in 2007 [9]. Over the same period, hypertension relative to the total reported outpatient diseases increased from 1.7% to 4.0% in all ages. In most regions, hypertension ranks as the fifth commonest cause of outpatient morbidity. However, in the Greater Accra Region of Ghana, hypertension moved from fourth to become second to malaria as the leading cause of outpatient morbidity in 2007 [10]. Stroke and hypertension are among the leading causes of admission and death. Hypertension is an important cause of heart and renal failure in Ghana [11,12]. Comprehensive reviews of studies on hypertension in Ghana are lacking. This systematic review was therefore undertaken to critically appraise existing studies on the prevalence of adult hypertension in Ghana, and its associated factors. The review should provide essential information to stimulate priority attention to cardiovascular diseases.

## Methods

### Study Area

**Figure 1:**
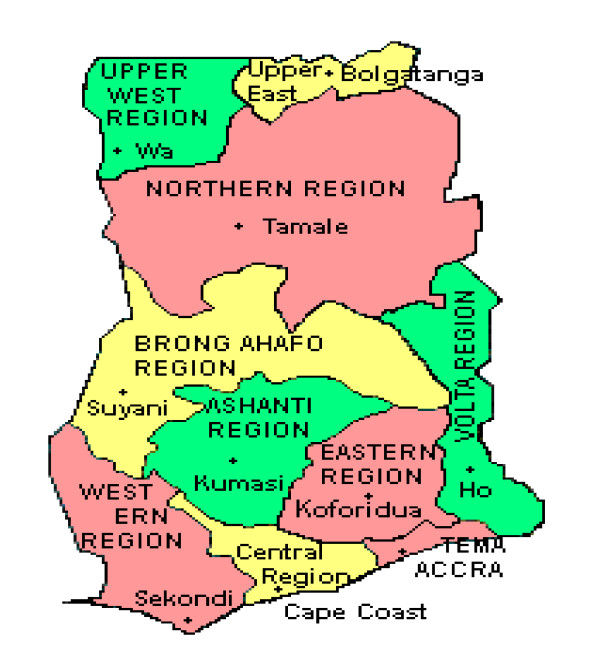
**Map of Ghana showing administrative regions**.

Ghana is located on the Atlantic Coast of West Africa 4 degrees north of the Equator. It occupies a land area of 238,537 km^2 ^and had a population of 23.5 million of which 56% is rural. Life expectancy at birth was 55 years for males and 60 years for females in 2000 [13]. The country is divided into ten administrative regions and 170 districts (Figure 1). Per capita total health expenditure as a percentage of GDP was 4.5% in 2003. Ghana ranked 135 out of 177 countries on the human development index in 2007 [14]. Fifty eight percent of the population lives less than 30 minutes of a public or private health facility, with geographical access being considerably better in the urban (78.5%) than in the rural populations (42.3%) [15]. The two largest cities Accra, the national capital and Kumasi, the regional capital of the Ashanti Region, have the highest concentration of doctors in the country. About 60% of persons who report ill or injured consult a health practitioner while 32% purchase medicines directly from pharmacy shops for their ailments [16].

### Data extraction

Potentially relevant papers on hypertension in Ghana were identified through a search of the PubMed and Google Scholar databases from 1970 to August 2009, supplemented by manual scan of the bibliographies of retrieved articles. Direct contact was made with some of the authors of included papers to locate some articles which were not accessible through HINARI or were unpublished. The key words used for the search were hypertension, blood pressure, cardiovascular and Ghana.

Studies included were those relating to adult non-pregnant human subjects aged 13 years and older published in English between January 1970 and August 2009. In addition, Master of Public Health student dissertations from the University of Ghana between 1996 and 2008 were reviewed and community-based prevalence studies on hypertension selected for analysis.

Published studies were excluded if they did not meet the above criteria or if they pertained to studies on patients. The abstracts of potentially relevant articles were reviewed out of which the full text articles of those deemed to be relevant were obtained for analysis. Eligible articles were assessed by the sole author of this paper. A standard data extraction sheet was used to extract information which included the study location, sampling strategy, sample size, sample demographic characteristics, prevalence, mean systolic and diastolic blood pressure, prevalence of hypertension and where available, the independent associated factors. Data on the awareness, treatment and control of those found or known to be hypertensive were obtained. Data quality issues assessed included sampling procedure, sample size estimation, response rate, selection bias, control of confounding, and quality control in blood pressure measurement. Differences in the methods of measuring blood pressure for each study were described rather than directly assessed against a gold standard. No pooled analysis was performed owing to the heterogeneity of study populations and the absence of datasets.

## Results

### Description of studies

**Figure 2:**
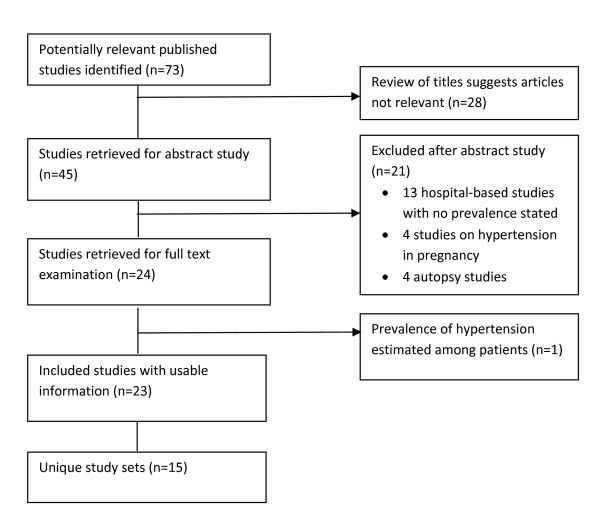
**Flowchart summarising search of published literature**.

The primary search of the database identified 68 individual papers and the hand search provided three additional papers. Google Scholar identified two additional papers. Thus, a total of 73 papers were identified for evaluation (Figure 2). Twenty four papers estimated prevalence of hypertension but one of them was among patients attending a blood pressure (BP) clinic and so was excluded [17]. Eventually, 15 unique published papers including two from Google Scholar [18,19] and one from conference proceedings [20] were included in the analysis of hypertension in Ghana. One set of studies involved a follow-up study of the original cohort after five years [20,21]. Another study conducted among public servants was complemented by a further study on a subset of sample which involved detailed cardiovascular investigations [22-24]. The original and subset study were reckoned as a single study. In addition to the published studies, three out of 350 MPH student dissertations were found to be relevant [25,26]. Of these, one had been published [27] and so overall, 17 unique published and unpublished papers were analysed (Table 1).

**Table 1:** Background characteristics of study sample and prevalence of hypertension by sex

Study references	Year of study	Location	Region	Population type	Participants	Sample size sex			Sample size residence		Response rate	Age in years		Prevalence ≥ 140/90			Crude Prevalence ≥ 160/95		
								
						Men	Women	Total	urban	rural		Range	mean	Men	Women	Total	Men	Women	Total
Pobee et al 1979 [22-24,74]	1972/1973	Accra-Tema	GAR	urban	public servants	5520	1380	6900			91.0	15 -64	nr	nr	nr	nr	9.8	4.3	8.9
Pobee et al 1977 [24,32]	early 1973	Danfa cluster of 20 villages^a^	GAR	rural	general pop.	809	861	1670	-	1670	97.5	16 -75+	nr^k^	nr	nr	nr	4.2	4.8	4.5
Ikeme et al 1978 [21,22,37,41]	Sept 1975 - May 1976	Mamprobi, Accra	GAR	urban	general pop.	1637	2108	3745	3745	-	73.0	15 -64	nr^l^	28.5%^m^	23.2%^m^	25.5%^m^	16.1	10.8	13.1
Chukwuemeka et al 1983 [20,74]	1981	Mamprobi, Accra	GAR	urban	general pop.	nr	nr	928	928	-	25.0	15 -64	nr	nr	nr	nr	nr	nr	13.0
Hesse 1998 [30]	Mar - Jun 1995	James Camp Prison in Accra^b^	GAR	urban	Male Prison Officers; prisoners	292^g^	-	292	292	-	officers 81.4 prisoners 95.3	Nr	officers = 39.8 ± 9.5; prisoners = 31.1 ± 10.3	nr	nr	nr	officers = 36.4; prisoners = 2.9	-	nr
Amoah 2003 [33]	1998	2 urban and 2 rural sites	GAR	Rural; urban	general pop.	1860	2873	4733	nr	nr	75	25 - 102	44.3 ± 14.7	27.6	29.5	28.3	14.7	17.4	16.2
Addo et al 2006 [27]	2001	4 sites, Ga district^c^	GAR	rural	general pop.	107	255	362	0	362	60-80% of villages	18 - 99	42.4 ± 18.6	24.1	25.9	25.4	nr	nr	15.2
Burket 2006 [35]	April 2002	Liati and Tokor, Kpando district	VR	rural	general pop.	66	221	287	0	287	99.0	17 -60+	41.8^h^	39.4	30.7	32.8	nr	nr	nr
Cappucio et al 2004 [36,75]	June 2001 - June 2002	6 semi-urban^d ^and 6 rural	AR	Rural; semi-urban	general pop.	385	628	1013	532	481	53.4	40 -75	54.7 ± 11.3	29.9	28.0	28.7	nr	nr	nr
Escalona et al 2004 [18]	Oct 2000 - Sept 2002	6 sites, Accra^e^	GAR	urban	general pop.	257	341	598	598	nr	nr	15 - 65+	nr	27.6	26.1	26.8	nr	nr	nr
Hill et al 2007 [29,44]	2003	Accra Metropolis	GAR	urban	general pop. of women	0	1328	1328	1328	-	41.5	18 -100	46.8 ± 18.0	-	48.0	48.0	nr	nr	nr
Agyeman et al 2006 [28,42]	2004	Kumasi and 4 villages	AR	Rural; urban	general pop.	787	644	1431	853	578	82-99	16 -50+	35.9	31.0	28.0	29.4	nr	nr	nr
Addo et al 2008 [31,76]	2005	7 ministries, Accra^f^	GAR	urban	civil servants	615	400	1015	1015	nr	82.7	25 -68	44.0 ± 10.1	31.7	28.0	30.2	nr	nr	nr
Owiredu et al 2008 [19]	Aug - Oct 2005	3 Pentecostal churches, Kumasi	AR	urban	Church goers	117	266	383	383	-	Nr	18 - 85	41.6 ± 13.4	SBP = 12.0; DBP = 9.4	SBP = 29.3; DBP = 25.6	SBP = 24.0; DBP = 20.6	nr	nr	nr
Kunutsor & Powles 2009 [34]	Feb - Apr 2007	Kassena-Nankana District	UER	rural	general pop.	207	367	574	-	574	95.7	15 - 65	37.8 ± 14.1			19.2			
Mensa-Wilmot 2003 [26]	2003	Kassena-Nankana District	UER	rural	general pop.	852	1166	2018	-	2018	Nr	15 -64	36.0 ± 14.6^i^	SBP = 5.6; DBP = 7.2	SBP = 3.8; DBP = 5.4	SBP = 6.2; DBP = 4.5	nr	nr	nr
Akufo 2008 [25]	2008	Kumasi metropolis	AR	urban	factory workers	251	49	300	300	-	Nr	17 -72	34.5^j^	22.7	18.4	22.0	nr	nr	nr

nr = not reported; GAR = Greater Accra Region; AR = Ashanti Region; UER = Upper East Region; VR = Volta Region; general pop. = general population

a = Danfa villages within 80 km of Accra; b = Low security prison; c = Sarpeiman, Opah, Ayikai Doblo, Amamoley in Amasaman sub-district; d = semi-urban sites (Tikrom, Appeadu, Duase, Apatrapa, Feyiase, Nwamase) up to 15 km from Kumasi, rural sites (Pemenase, Edwenase, Domeabra, Ofoase, Atia, Dumakwai) up to 40 km from Kumasi in the Kumasi and Ejisu-Juaben districts; e = communities are Teshie, Sakaman, Awoshie, Onyinase, Dansoman, Mamprobi; f = out of 26 ministries; g = 88 Prison officers and 204 prisoners; h, i, j = median age 40, 35, 39 years respectively; k = 63.8% aged < 45 years; l = 36.5% of sample aged 15-25 years; 5.8% aged 55-64 years; m = based on 2,001 men and 2,702 women

The selected studies were conducted between 1973 and 2008 in four out of Ghana's ten regions - Greater Accra (10), Ashanti (4), Upper East (2) and Volta Regions (1). Nine of the studies were conducted in urban populations, five in rural populations and three in mixed populations. The study populations were varied and included general population, volunteers, public servants, factory workers and church worshippers. One study selected subjects from churches, schools and banks [28]. Except for one study which involved only women [29] and another which involved only men [30], the rest of the studies involved both sexes. The proportion of women in the studies ranged from 16% to 100%. In 14 studies involving both sexes, ten enrolled more females than males and up to 77% of study subjects were females (Table 1). As expected, males were generally predominant in study populations such as civil servants, factory workers and prison officers [23,25,31].

As with the sex composition, the age profile of study subjects was heterogeneous, ranging from 15 to 102 years. The mean age of participants was less than 40 years in six studies, between 40 and 50 years in six studies and around 55 years in one study. Although the mean ages were not provided for four studies, it is most likely that the mean age was less than 40 years in three of these studies. In one of these studies, 64% of the subjects were younger than 45 years [32] and in another 37% were aged 15 to 25 years (with only 6% aged 55-65 years) [21]. In some studies, there was an attempt to oversample older subjects to compensate for their smaller size within the general population [29].

A total of 26,649 different adults were involved in the reviewed studies (Table 1). The sample size ranged from 287 to 6,900 adults. It was more than 1,000 in 8 studies and less than 500 in five studies. Except for three studies, the sample size was either not based on any calculation or this was not reported. Thirteen studies employed rigorous sampling strategy at all levels of sampling with well defined sampling frames at the household or institutional level through the use of household census [20,21,26,29,32-34], electoral registers [27] or staff registers [23,25,28,30,31]. Three studies lacking defined sampling frames examined interested volunteers on a market day [35], Christian worshippers attending church services or persons in every other house in a rural village [28].

The response rate ranged from 25% to 98% between studies (Table 1) [20,32]. Only three studies reported response rates less than 60% [20,29,36]. In the study with the lowest response rate, only 25% of the original members of a cohort could be located five years later. Within studies, the response rate differed between the study groups, by sex and by location. For example, 40% to 80% of persons in 12 villages invited to take part in the study agreed to do so [36]. Only five studies described reasons for non-response or characteristics of non-responders [20,24,27,36,37]. Non-response was due to feeling healthy, feeling ill, temporary absence, fear of premature retirement if found to be a hypertensive public servant, interference with time at work, and lack of interest.

### Blood pressure measurement

Nearly all studies reported significant investments to ensure good quality BP readings. These investments included use of experienced nurses, periodic re-certification of personnel, confirmatory readings by supervising physician and the use of standardized measurement protocols. Reference methods guiding blood pressure measurement and analyses that were mentioned by individual studies were WHO MONICA [34,38], the Joint National Committee on Prevention, Detection, Detection, Evaluation, and Treatment of High Blood Pressure (JNC 6 or 7) [31,33], the WHO and International Society of Hypertension Guidelines [31,39,40], American Heart Association [19], other WHO guidelines [7,20,32], video-tutored course (Shared Care, Torrance, California) [27,33], other published studies [29] and unspecified standard methods [18]. One study trained the blood pressure measurement team to achieve inter-observer variation of less than 4 mmHg [35].

Eleven studies (65%) employed manual mercury sphygmomanometers while six studies (35%) used electronic BP monitors, usually the Omron BP monitor (Table 2). Of the 9 studies describing the cuff size used for the BP measurement, nearly all reported using more than one cuff size to suit the small and large arms. One study used one cuff size for the arms of all subjects while another used a wrist BP monitor [26]. Up to 12 trained personnel, usually nurses, measured the blood pressures in each study. In auscultatory measurements, the first and the fourth [21] or fifth [27,30,33] Korotkoff phase sounds corresponded to the systolic (SBP) and diastolic blood pressure (DBP) respectively. Blood pressure was generally taken on the right arm of seated patients and in one case, also in supine subjects [29]. Preparation commonly involved an initial rest period of at least five minutes and occasionally, prohibition of smoking [25,33].

**Table 2:** Blood pressure measurement techniques in epidemiological studies on hypertension in Ghana

Study references	Personnel taking BP	No. of visits	Interval between visits	Frequency of readings per visit	Initial rest time (mins)	Interval between multiple readings	Reading used in analysis	cuff size	Posture	Part of body on which BP taken	Device
Pobee et al 1979 [22-24,74]	nr	1 -2; If 2^nd ^BP reading higher than that defined for age, BP re-taken at a later visit	24 hours	1 - 3	nr	10 mins	1^st ^or 3^rd ^BP reading	nr	seated	right arm	manual
Pobee et al 1977 [24,32]	2 field staff	1	-	3	5-10	nr	Mean of three readings	14 cm wide cuff	seated	right arm	manual
Ikeme et al 1978 [21,22,37,41]	trained nurses	1	-	1 - 3; BP repeated twice if BP > 160/95 mmHg	5	10 mins	Initial BP	two cuff sizes 12 × 10 cm, 14 × 17 cm	seated	right arm	Manual
Chukwuemeka et al 1983 [20,74]	trained nurses	1	-	As in Ikeme et al 1978[21]	5	nr	nr	two cuff sizes 12 × 10 cm, 14 × 17 cm	seated	right arm	manual
Hesse 1998 [30]	4 medical officers	1 - 2; BP re-taken at a later visit if BP > 140/90 mmHg	1 month	3	5	30 secs	Mean of latter 3 readings	appropriate cuff size	seated	right arm	manual
Amoah 2003 [33]	3 nurses	1	-	2	≥ 10	1 min	Mean of two readings	appropriate cuff size	seated	right arm	manual
Addo et al 2006 [27]	2 nurses	1	-	2	≥ 10	1 min	Mean of 2 readings	appropriate cuff size	seated	Right arm	manual
Burket 2006 [35]	Nurses, confirmed by physician if raised BP	1	-	1 - 2; BP repeated only if 1st reading is raised	nr	On average, 26.5 mins	nr	nr	seated	arm	manual
Cappuccio et al 2004 [36,75]	Doctors, nurses, clerks	1	-	3	≥ 5	1 min	Mean of 2^nd ^and 3rd readings	appropriate cuff size	seated	arm	electronic OMRON HEM705CP
Escalona et al 2004 [18]	Nurses	1	-	nr	nr	nr	nr	nr	seated	arm	Manual
Hill et al 2007 [29,44]	2 trained nurses	1	-	2	nr	nr	nr; both readings had to be raised	two cuff sizes	1303 seated 1253 supine	arm	Manual
Agyeman et al 2006 [28,42]	Trained staff	1	-	2	5	≥ 5 mins	Mean of two readings	appropriate cuff size	seated	right arm	electronic - Omron M5-I
Addo et al 2008 [31]	Trained interviewers	1 - 2, BP repeated at a later visit if initially > 140/90 mmHg without treatment	3 weeks	1-3	≥ 10	1 min	Mean of 2nd & 3rd readings	appropriate cuff size	Seated with feet flat on floor	right arm	electronic - Omron M5-I
Owiredu et al 2008 [19]	Trained personnel	1	-	2	> 5	5 mins	Mean of two readings	nr	seated	Left arm	Manual
Kunutsor & Powles 2009 [34]	6 trained personnel	1 - 2 BP re-taken in consenting sub-sample at a later visit	2 weeks	1 - 2	nr	nr	Mean of two readings	nr	seated	right arm	electronic - OMRON MX3 Plus
Mensa-Wilmot 2003 [26]	nurses	1	-	3	≥ 5	≥ 5 mins	Mean of 2nd and 3rd readings	nr	seated	wrist	electronic OMORON R5-I wrist digital
Akufo 2008 [25]	Nurse and 2 field assistants	1	-	3	≥ 30	5 mins	Mean of the 3 readings	nr	seated	right arm	electronic OMRON M4-I digital

Nr = not reported

Except for four studies, all studies reported taking BP during a single visit. Studies which measured BP at two visits, repeated the measurements one day [23], 3 weeks [31] or one month [30] later for those whose BPs were greater than 140/90 mmHg during the initial visit [30,31]. In one study, repeated measurements were done for 16% of study subjects 2 weeks later in order to correct a regression dilution effect arising because baseline BP tends to underestimate the usual BP [34]. The repeated measurements permitted a regression dilution ratio to be calculated and used to adjust the baseline casual BP to estimate the usual BP.

During a single visit, the BP was measured one to three times between different studies (Table 2). In studies with a single BP reading, measurement was only repeated in those with an initially raised reading [20,21,31,35,41]. Multiple BP readings were separated by intervals of 30 seconds to 27 minutes in different studies [30,35]. The average of two [19,27,33,42] or three [25,30,32] readings were usually used in the analysis, with some studies ignoring the first reading and using the mean of the second and third reading [26,31,36]. The Mamprobi 1975 study analysed the initial of the two repeated measurements in those with BP higher than 160/95 mmHg [21,41].

### Prevalence of hypertension

The five studies conducted up to 1995 used a threshold of 160/95 mmHg for hypertension while 13 studies conducted later used a threshold of 140/90 mmHg. Most of the latter studies also considered self-reported hypertension as hypertensives in addition to those with raised BP [18,27,31,33]. However, some studies restricted their diagnosis of hypertension to only the blood pressure readings [19,34,35,43]. One study specifically excluded subjects who reported being hypertensives or diabetics from the analysis [19].

Most studies reported a crude prevalence of hypertension between 25% and 48%, using the newer threshold of 140/90 mmHg (Table 1). The Women's Health Study of Accra (WHSA) reported a crude prevalence of 54.6% among 1,303 women in upright position [44]. Only four studies reported a prevalence of less than 20%. Regardless of the cut-off used, most studies reported a higher prevalence among men than among women though differences were frequently small (< 4 percentage points) and where assessed, not statistically significant [36]. Only four of 15 studies reported a higher prevalence in women [19,27,32,33]. The widest sex disparities in prevalence were observed in the rural Kpando district [35] and in the early urban studies of Accra [21,23,24]. In one study, the prevalence in men was more than twice that in women [23]. The pattern of sex differences in the prevalence of hypertension remained after adjusting for age [27,31,33].

In mixed populations, the prevalence of hypertension was higher in urban than in rural populations (Table 3). In four of six rural populations, prevalence of hypertension (BP ≥ 140/90 mmHg) was 24% or higher [27,28,35,36]. It is striking that in the other two studies with lower prevalence, both conducted in the same rural district in the Upper East Region, there was a three-fold disparity in the prevalence of hypertension [26,34].

**Table 3:** Mean systolic and diastolic blood pressure and urban-rural prevalence of hypertension

Study references	Population type	Mean Age in years	Prevalence ≥140/90		Crude Prevalence ≥160/95		Mean systolic BP			Mean diastolic BP		
			
			urban	rural	urban	rural	Male	Female	Total	Male	Female	Total
Pobee et al 1979 [22-24,74]	Urban public servants	nr	8.0	nr	8.0	-						
Pobee et al 1977 [24,32]	rural	nrk	nr	nr	-	4.5	123.8 ± 19.7	122.0 ± 21.0	122.5	69.2 ± 13.5	68.8 ± 12.7	69
Ikeme et al 1978 [21,22,37,41]	urban	nrl	25.5	-	13.1	-	127.7 ± 18.7	124.6 ± 20.2	125.9 ± 9.6	80.5 ± 13.0	78.9 ± 13.9	79.6 ± 13.6
Chukwuemeka et al 1983 [20,74]	urban	nr	nr	nr	13.0	-	133.6 ± 19.9	136.7 ± 22.9	135.2 ± 21.4	85.5 ± 11.3	86.1 ± 13.4	85.8 ± 12.4
Hesse 1998 [30]	Urban Prison	officers = 39.8 ± 9.5; prisoners = 31.1 ± 10.3	nr	nr	officers = 36.4; prisoners = 2.9	-	officers = 140.9 ± 25.1; prisoners = 123.3 ± 14.4	-	nr	officers = 91.3 ± 16.7; prisoners = 77.2 ± 10.4	-	nr
Amoah 2003 [33]	Rural; urban	44.3 ± 14.7	nr	nr	nr	nr	129.0 ± 22.2	128.9 ± 26.7	128.9 ± 25.0	75.1 ± 13.0	74.7 ± 14.1	74.9 ± 13.7
Addo et al 2006 [27]	rural	42.4 ± 18.6	-	25.4	nr	15.2	125.4 ± 20.9	128.5 ± 27.6	127.5 ± 25.8	74.5 ± 14.2	73.9 ± 14.4	74.0 ± 14.3
Burket 2006 [35]	rural	41.8 h	-	32.8	nr	nr	nr	nr	nr	nr	nr	nr
Cappucio et al 2004 [36,75]	Rural; semi-urban	54.7 ± 11.3	32.9	24.1	nr	nr	126.3 ± 24.4	125.1 ± 27.0	125.5 ± 26.1	75.8 ± 13.7	73.5 ± 13.5	74.4 ± 13.6
Escalona et al 2004 [18]	urban	nr	26.8	nr	nr	nr	nr	nr	nr	nr	nr	nr
Hill et al 2007 [29,44]	Urban women	46.8 ± 18.0	48.0	-	nr	nr	-	upright: 139.4 ± 27.5; supine 141.2 ± 28.0 mmHg	upright: 139.4 ± 27.5; supine 141.2 ± 28.0 mmHg	-	upright: 86.4 ± 15.1; supine 87.6 ± 15.3 mmHg	upright: 86.4 ± 15.1; supine 87.6 ± 15.3 mmHg
Agyeman et al 2006 [28,42]	Rural; urban	35.9	31.1	27.0	nr	nr	nr	nr	nr	nr	nr	nr
Addo et al 2008 [31]	Urban civil servants	44.0 ± 10.1	30.2	-	nr	nr	131.5	121.5	128.5	80.0	77.0	79.0
Owiredu et al 2008 [19]	Urban church attendees	41.6 ± 13.4	SBP = 24.0; DBP = 20.6	-	nr	nr	117.1 ± 15.7	126.4 ± 21.8	123.6 ± 20.5	72.9 ± 10.3	77.7 ± 13.5	76.2 ± 12.8
Kunutsor & Powles 2009 [34]	rural	37.8 ± 14.1		19.2			124.3 ± 18.7	122.1 ± 22.0	122.9 ± 20.9	69.9 ± 12.1	72.1 ± 12.4	71.3 ± 12.3
Mensa-Wilmot 2003 [26]	rural	36.0 ± 14.6i	nr	SBP = 6.2; DBP = 4.5	nr	nr	mean SPB in 45+y group = 119.9 ± 17.6	mean SPB in 45+y group = 116.1 ± 15.8	All ages = 113; mean SPB in 45+y group = 117.6 ± 16.6	Mean DPB in 45+y group = 78.3 ± 13.5	Mean SPB in 45+y group = 74.5 ± 10.7	All ages = 73.7; Mean DPB in 45+y group = 76.0 ± 18.0
Akufo 2008 [25]	urban factory workers	34.5j	22.0	-	nr	nr	nr	nr	nr	nr	nr	nr

Nr = not reported

### Factors associated with hypertension

Within studies, the prevalence of hypertension increased with increasing age although the gradient was not always monotonic [23,37]. The age-standardized prevalence [31,45] or the weighted prevalence [29] was similar or lower than the crude prevalence where these were provided. For instance, the 48% crude prevalence of hypertension in the Women's Health Study of Accra compares with a weighted prevalence of 40% [29]. Hypertension also increased with increasing body mass index.

Between studies with available data, the mean systolic BP was in the range 113.0-140.9 mmHg and the diastolic BP in the range 69.0-86.4 mmHg (Table 3). There was weak correlation between the mean age and the mean SBP (*R *= 0.20, p = 0.29) or the mean DBP (*R *= 0.05, p = 0.45). The mean systolic and diastolic blood pressures were higher in urban than in rural areas.

The age-specific mean blood pressures varied between studies and were higher in the older age groups (Table 4). The mean age-specific blood pressures were generally higher in men in the younger age groups and higher in women from 55 years. The widest disparities in mean age-specific systolic and diastolic blood pressures were observed in the older age groups. The lowest age-specific diastolic pressures were recorded by the rural Danfa study [32] while the highest DBPs were recorded by the urban Mamprobi cardiovascular study [20], both studies conducted in the 1970s. The latter study [20] also recorded the highest age-specific systolic BPs whereas the lowest age-specific BPs varied inconsistently between studies.

**Table 4:** Mean age-specific systolic and diastolic blood pressure in selected studies

Study ref	Blood Pressure	15-24			25-34			35-44			45-54			55-64			65-74			75 +		
		
		M	F	T	M	F	T	M	F	T	M	F	T	M	F	T	M	F	T	M	F	T
Pobee et al 1977 [24,32]^a^	SBP	120.9	113.8	117.2	122.1	113.4	117.3	121.7	118.1	119.8	120.3	127.4	123.7	127.8	130.8	129.2	130.2	133.5	131.9	143.4	151.6	147.2
	DBP	60.6	62.7	61.7	69.1	64.0	66.3	71.5	70.5	71.0	72.7	75.8	74.2	74.3	75.8	75.0	74	74.1	74.1	76.1	78.1	77.0
Chukwuemeka et al 1983 [20,74]^b^	SBP	116.4 ± 13.4	115.1 ± 13.2	-	117.6 ± 14.4	118.9 ± 15.5	-	126.9 ± 20.4	127.7 ± 23.2	-	137.8 ± 19.8	147.1 ± 31.1	-	149.1 ± 22.3	150.1 ± 24.5	-	153.5 ± 29.0	161.0 ± 29.8	-	-	-	-
	DBP	74.9 ± 8.7	73.8 ± 8.2	-	76.6 ± 9.1	73.8 ± 11.4	-	83.6 ± 13.7	85.3 ± 13.6	-	88.9 ± 11.3	94.0 ± 17.0	-	95.1 ± 12.1	93.0 ± 12.9	-	93.7 ± 12.6	96.6 ± 17.4	-	-	-	-
Amoah 2003 [33]	SBP	-	-	-	120.7 ± 13.5	115.3 ± 15.3	-	123.1 ± 16.7	122.6 ± 20.0	-	131.7 ± 23.0	133.9 ± 26.3	-	144.0 ± 29.4	143.9 ± 28.2	-	128.1 ± 21.6	127.7 ± 25.7	-	-	-	-
	DBP	-	-	-	70.0 ± 9.7	68.2 ± 10.2	-	74.5 ± 12.5	74.3 ± 13.0	-	79.2 ± 13.9	78.7 ± 13.7	-	75.5 ± 13.3	79.8 ± 15.0	-	74.6 ± 12.7	74.2 ± 13.8	-	-	-	-
Cappucio et al 2004 [36,75]^c^	SBP	-	-	-	-	-	-	120.9 ± 18.6	108.7 ± 16.8	-	124.6 ± 22.2	122.8 ± 25.4	-	130.0 ± 25.5	129.7 ± 28.1	-	130.0 ± 30.0	138.1 ± 28.0	-	-	-	-
	DBP	-	-	-	-	-	-	75.1 ± 13.9	67.7 ± 11.3	-	76.6 ± 13.6	74.3 ± 13.3	-	77.3 ± 14.4	75.6 ± 13.6	-	73.4 ± 12.9	75.2 ± 14.2	-	-	-	-
Addo et al 2008 [31]^d^	SBP	-	-	-	125.0	113.5	119.0	127.5	117.5	125.0	135.5	130.8	134.0	140.3	131.8	137.0	-	-	-	-	-	-
	DBP	-	-	-	72.5	69.0	71.3	77.5	76.0	76.5	84.5	83.5	84.0	85.0	79.8	84.5	-	-	-	-	-	-
Owiredu et al 2008 [19]^e^	SBP	-	-	109.5 ± 10.8	-	-	113.8 ± 11.6	-	-	121.7 ± 18.4	-	-	127.2 ± 18.8	-	-	139.1 ± 25.8	-	-	150.0 ± 20.6	-	-	-
	DBP	-	-	67.7 ± 7.7	-	-	70.8 ± 8.7	-	-	76.2 ± 12.8	-	-	80.1 ± 12.4	-	-	81.4 ± 15.5	-	-	85.7 ± 14.0	-	-	-
Kunutsor & Powles 2009 [34]	SBP	123.3 ± 13.0	113.5 ± 11.4	117.5 ± 13.0	124.1 ± 19.2	112.5 ± 11.0	116.7 ± 15.5	124.2 ± 16.2	123.1 ± 21.3	123.5 ± 19.3	124.4 ± 24.5	130.8 ± 25.9	129.0 ± 25.5	126.2 ± 23.7	134.0 ± 28.4	131.5 ± 27.1	-	-	-	-	-	-
	DBP	61.9 ± 6.6	65.6 ± 8.4	64.1 ± 7.9	69.9 ± 12.6	67.9 ± 9.2	68.6 ± 10.6	71.8 ± 9.9	76.1 ± 13.2	74.3 ± 12.1	75.5 ± 14.8	78.3 ± 13.7	77.5 ± 14.0	75.2 ± 12.2	74.0 ± 12.1	74.4 ± 12.1	-	-	-	-	-	-

a = lowest age group 16-24 years; b = highest age group > 65 years; c = lowest age group 40-44 years; d = median BP values, highest age group > 55 years; e = lowest age group 19-24 years, highest age group > 65 years

In bivariate analyses in which this was assessed, the mean SBP, mean DBP or prevalence of hypertension increased with increasing age [7,19,20,27-29,31-34,36,37]. Similarly, these same parameters tended to increase with increasing BMI [18,19,21,27,31,34,42,44].

Multivariate analyses to adjust for the effects of potential confounders were performed by six studies [19,27,28,30,31,34]. Factors independently associated with SBP or DBP in various combinations in the different studies were older age, menopause before age 50 years, no formal education, overweight or obesity, urban residence, land ownership, and male sex [27-31,34]. On the other hand, having completed six years of education [27], alcohol consumption [28] and time of blood pressure measurement [34] were inversely associated with blood pressure. In the study reporting the highest association with BMI, obese persons had 6.9 (95% CI 1.7, 28.2) times the odds of having hypertension as those with normal BMI.

In multiple linear regression analysis, both age and BMI were positively and independently associated with SBP and DBP in females, while age was positively and independently associated with DBP in males in the rural Kassena Nankana district [34]. Findings were not always consistent between studies. In Kumasi, abnormal waist circumference was associated with higher odds for SBP or DBP compared to those with normal waist circumference, with the difference barely reaching statistical significance. However, the association between abnormal WHR, BMI and waist to height ratio and systolic or diastolic hypertension was not statistically significant [19].

### Detection, treatment and control

Detailed analysis of the awareness, treatment and control of hypertension was available for seven studies (Table 5). Of the hypertensives identified in these studies, only 22%-54% were aware of their condition, 7%-31% were on treatment and 0%-13% had their blood pressures controlled. Between studies, the proportion of hypertensives aware, on treatment and who had controlled blood pressure in urban populations was not markedly different from that in rural populations. The most favourable awareness, treatment and response was observed among civil servants in Accra [31] and the worst among public servants in Accra in 1973 [22]. Although the differences in study populations hinder direct comparison, there appeared to be a trend towards improved awareness, treatment and control (Table 5) from the mid-1970s to the late 2000s.

**Table 5:** Awareness, Treatment and Control of Hypertension

Study ref	Total no. Hypertensives	Among total number of hypertensives:			Aware %	Treatment %	Control %
					
		**Aware**^**a**^	**Treatment**^**b**^	**Control**^**c**^			
Pobee et al 1979 [22-24,74]	540	130	39	20	24.1	7.2	3.7
Hesse 1998 [30]	38	14	5	0	36.8	13.2	0.0
Amoah 2003 [33]	1337	458	243	49	34.3	18.2	3.7
Addo et al 2006 [27]	93	30	15	5	32.3	16.1	5.4
Cappucio et al 2004 [36,75]	291	64	33	8	22.0	11.3	2.7
Agyeman et al 2006 [28,42]	421	143	118	26	34.0	28.0	6.2
Addo et al 2008 [31]	307	166	96	39	54.1	31.3	12.7

^a^Hypertensives who reported having ever been informed by a health professional of a diagnosis of hypertension were considered to be aware of their condition.

^b^Hypertensives who reported taking recognized antihypertensive medication were considered to be on treatment.

^c^Hypertensives on medication whose blood pressure measured during the study was less than140/90 mm Hg were considered to have controlled blood pressure controlled.

Note that the percentages in columns 6-8 use the total number of hypertensives as a common denominator

In one study, age group older than 35 years relative to the 16-34 years was independently associated with being aware of hypertension after adjusting for age group, sex, locality, educational level, occupation, BMI, land ownership, smoking and alcohol consumption [28]. Older age from 50 years, trading and overweight were independently associated with being on drug treatment while trading was independently was associated with adequate blood pressure control.

## Discussion

Pobee *et al *[23] described an epidemic of hypertension in Ghana in 1979. This review has demonstrated that the epidemic has persisted or increased. With a conservative estimate of 15.8 million adults aged 15 years or older in 2008 , 48% urbanization [46], hypertension prevalence of 25% in urban and 20% in rural populations, it is estimated that, at least, 3.5 million adults have hypertension. In comparison, 236,151 adults were estimated with living with HIV and AIDS in Ghana in 2008 [47]. Yet, national response to hypertension is considerably much weaker.

This review is the most comprehensive analyses of prevalence studies on hypertension in Ghana to date. Several issues of international relevance emerge from this review. Hypertension is a significant problem not only in urban populations but also in poorest and leanest rural populations [34]. Also, even in relative young populations with a mean age of 36 years, 29% are hypertensive [28]. These observations should help dispel the prevailing myth that hypertension is a major problem in only affluent or elderly populations [48]. With the inevitable increase in urbanization, the psychosocial distress associated with migration, dietary and physical activity changes, hypertension will probably persist or worsen [49]. Currently, self-reported fruit and vegetable consumption intake Ghana is among the lowest in the world [50] and the frequency of obesity has been increasing [51] reaching up to 35% in some populations [29]. Increase in the prevalence of such risk factors contributed to a significant increase in hypertension in Tanzania over a relative short period of 11 years [5].

The estimated prevalence of hypertension is consistent with reported prevalence in other parts of Africa [4,52]. For instance, the prevalence (≥ 140/90 mmHg) in the neighbouring cities of Abidjan in 2005 and Cotonou in 2007 was 21.7% and 27.3% respectively [53]. Higher prevalence was reported in semi-urban Nigeria (37%) [52], Burkina Faso (40%) [54], and Niger (42%) [53]. As in this review, most studies reported a higher prevalence in urban than in rural areas [4,55,56]. However, some countries did not find any urban-rural difference in hypertension prevalence [57,58].

Several studies in Africa show minimal sex differences in prevalence of hypertension [4,57]. As in Ghana, some countries report a higher prevalence among men [53,59] while others report the converse [52,60]. Male or female preponderance of hypertension could differ in the same study subjects depending on the threshold used [52].

Hypertension often occurs with co-morbidities such as obesity, dyslipidaemia and diabetes [19], thereby increasing risk of its complications. Urgent action is hampered by low political will, limited interest from development partners, low funding and low public awareness in Ghana. The pervasive low awareness, even among the relatively educated civil servants, should stimulate health authorities and civil society to campaign for both provider-led and community-driven periodic medical check-ups. Health practitioners should be sensitized to opportunistically check the blood pressures of adults who visit the clinic for any reason. Taking a cue from 'Know your HIV status' campaigns throughout Africa, health authorities could similarly embark on 'Know your blood pressure' campaigns, for example by designating one month for this each year and include blood pressure screening in workplace programmes.

Awareness of hypertension is better in Ghana than in countries such as Eritrea, Burkina Faso, Cameroon and The Gambia where only 17%-23% of hypertensives were known hypertensives [54,56-59]. Control of hypertension in most parts of Africa is low; a mere 2% or lower in Tanzania [55] and Cameroon [59]. Low compliance to treatment and subsequent default from treatment in Ghana is due to high cost of drugs, ready access to herbal treatment, misconceptions that hypertension in curable, and inadequate counselling [17,61,62]. Health care workers often lack access to basic, practical information to equip them to provide adequate care [63]. The absence of national treatment guidelines for hypertension in Ghana contributes to the multiplicity of drug regimens [64]. Lifestyle interventions are not routinely provided as part of the management of hypertension or in primary care. Noncompliance with therapy and recourse to alternative medicines are a major barrier that could contribute to the persistent poor blood pressure control among hypertensives [62].

A number of opportunities that have recently emerged should favour such lifestyle and other interventions in Ghana. These include the introduction of a National Health Insurance Scheme (NHIS) in 2006 that could reduce financial barriers to treatment, a paradigm shift in national policy towards health promotion in 2008 and a planned passage of a draft bill on tobacco control. Reducing salt intake has been shown to be feasible and beneficial to reduced population systolic BP in Ghana [65]. Legislation is needed to compel the food industry to reduce salt content of processed foods [66]. The changing perception among women to leaner body shape could motivate behaviour changes [43].

This review has also highlighted several methodological differences in terms of the number of visits for pre-diagnosis blood pressure assessment, interval between visits, interval between BP measurements, site of BP measurements, choice of cuff size, and choice of readings to analyse. The differences in the approaches to blood pressure measurement are well acknowledged in the literature [67,68]. Factors such as talking, acute exposure to cold, recent ingestion of alcohol, incorrect arm position, and incorrect cuff size have been shown to affect readings by more than 5 mmHg [68]. Attention to such details is not always evident in blood pressure studies from Africa.

The WHO recommends using the average of three blood pressure readings at one visit in risk factor surveys [69], although single visit measurements could result in an overestimation [70]. Interestingly, a recent study showed that the first of five office BP readings by a trained nurse using a manual sphygmomanometer effectively predicted the presence of a wide range of markers of target organ damage in a standardized ambulatory BP monitoring, as did the average of all five readings measured at 2-3 minute intervals [71]. On the whole, recent published guidelines from leading professional institutions provide adequate guidance on the measurement of blood pressure in different settings (e.g. office, home, clinic) [72,73]. Proper training and supervision is required to ensure that these guidelines are strictly followed in prevalence studies.

Study limitations such as the use of volunteers [18], non-random selection of participants [18], low response rates [20,44] and the use of sub-populations could limit the generalizability of the findings. The small sample size in many studies reduces the precision of several estimates and reduces the power of the study to detect differences between persons or geographical units. The three-fold difference in the estimated prevalence of hypertension in the Kassena-Nankana district deserves further study [26,34]. The consistently higher SBP and DBP recorded by one particular study [20] suggests a probable inter-observer team error between studies. A further weakness is that virtually all the studies are one-off cross-sectional studies thereby denying follow-up opportunity to monitor trends and changes in risk factor. The only cohort study in this series suffered a large loss to follow up after five years of about 75%, as to limit comparison between the two time periods [20,21]. Ghana therefore needs more surveillance studies that are repeated every four or five years, as recommended by the WHO [69]. Further studies should build on strengths of previous studies such as use of representative samples, measuring BP at more than one visit, quality control measures for blood pressure measurement, assessing complications and co-morbidities and the behaviour of persons with hypertension. These studies could be supplemented by qualitative studies that help to identify best approaches to improve awareness, treatment and control of hypertension.

## Conclusions

Notwithstanding the variations in methods between studies, this review has demonstrated a consistently high prevalence of adult hypertension in urban and rural areas of Ghana. The awareness, treatment experience and effective control of hypertension are low. Recent opportunities to control hypertension in Ghana have emerged which could facilitate prevention, detection and care of hypertension.

## Competing interests

The author declares that he has no competing interests.

## Authors' contributions

WKB is responsible for all aspects of the study including the study concept, data collection, interpretation of results and drafting, revision and finalization of the paper.

## Pre-publication history

The pre-publication history for this paper can be accessed here:

http://www.biomedcentral.com/1471-2458/10/418/prepub
